# Sleep Quality and Professional Burnout in Clinical Nurses: A Cross-Sectional Study

**DOI:** 10.3390/healthcare13212727

**Published:** 2025-10-28

**Authors:** Marius Baranauskas, Ingrida Kupčiūnaitė, Jurgita Lieponienė, Rimantas Stukas

**Affiliations:** 1 Faculty of Biomedical Sciences, State Higher Education Institution Panevėžys College, 35200 Panevėžys, Lithuania; ingrida.kupciunaite@panko.lt (I.K.); jurgita.lieponiene@panko.lt (J.L.); 2 Department of Public Health, Institute of Health Sciences, Faculty of Medicine, Vilnius University, 01513 Vilnius, Lithuania; rimantas.stukas@mf.vu.lt

**Keywords:** burnout, depersonalization, emotional exhaustion, healthcare, mental health, nurses, sleep quality

## Abstract

**Background/Objectives**: Healthcare workers often experience chronic psychological stress, which may affect up to 71% of nurses, leading to mental outcomes, namely, depressive symptoms and a chronic state of physical and emotional depletion followed by burnout syndrome. Emotional exhaustion, depersonalization and poor personal accomplishment are three core features responsible for the development of burnout. Given sleep quality as a mediator is likely to play a key role in forecasting the potential impingement of burnout both directly and indirectly, this cross-sectional study aimed to explore any possible association between sleep disorders and burnout in a cohort of Lithuanian clinical nurses. **Methods**: During a six-week period in October–November 2024, a total of 269 female nurses ranging between 22 and 67 years old were recruited for a cross-sectional study. The Pittsburgh Sleep Quality Index (PSQI) tool and the Maslach Burnout Inventory (MBI) were applied to assess the level of subjective sleep quality over the last month and the self-perceived occupational burnout experienced by clinical nurses, respectively. **Results**: This study highlighted a worrying proportion of nurses found to be at an increased risk of occupational burnout syndrome after more than 60% of nurses had experienced the symptoms of emotional exhaustion and depersonalization. A similar proportion of nurses was exposed to the risk of sleep disorders, which, as a potential trigger, played an important role in maintaining burnout syndrome. More specifically, the global PSQI score was related to the expression of depersonalization (β 0.5, 95% confidence interval (CI) 0.2; 0.9, *p* = 0.002, *R*^2^ = 0.27). The higher levels of both emotion exhaustion (β 2.5, 95% CI 1.5; 3.5, *p* < 0.001, *R*^2^ = 0.26) and depersonalization (β 1.9, 95% CI 0.8; 3.0, *p* = 0.001, *R*^2^ = 0.28) were associated with perceived daily disturbances (in terms of sleep disturbances and daytime dysfunction) in nurses. **Conclusions**: Healthcare professionals should focus further attention on reducing high-level depersonalization expression and potential risk factors, namely sleep disturbances and daytime dysfunction associated with this burnout symptom in a population of clinical nurses. Therefore, by targeted integration of efficient sleep interventions, healthcare institutions could promote employee-friendly workplaces, and, eventually, improve not only the indicators of burnout syndrome but also nurses’ performance and patient safety as well as satisfaction with perceived nursing care.

## 1. Introduction

Sleep is among the essential physiological demands of humans. Poor sleep quality can result in negative physical and psychological conditions triggering several harmful effects because of both abnormal sleep levels and poor sleep quality [1]. Also, sleep plays an important part in psychomotor learning, memory building and stabilization, as well as in the immune response, cardiovascular and hepatic metabolism [2]. Sleep disorders occur through dyssomnias, i.e., insomnia or hypersomnia, which are associated with the disturbances of a normal circadian rhythm, which, in turn, may result from shift work or other reasons related to jet-lag syndrome, various pathological conditions or altered environmental circumstances [3,4,5]. Although life expectancy and the incidence of noncommunicable diseases, such as mental illnesses [6,7], have increased worldwide, the demand for nurses has also steadily increased and the number of existing nurses is not able to meet a rising demand. In this context, female nurses represent an increased risk cohort for metabolic disorders and circadian rhythm abnormalities. Research has confirmed that inadequate sleep is adversely affecting nurses’ mental health [8,9] as well as triggering health problems related to diabetes and cardiovascular diseases [10,11]. Thus, when sleep disorders have become a predominant public health concern due to an increased workload, the influence of sleep quality ought to be further researched [12].

It should be highlighted that nurses have been weighed as occupying a position in which they are exposed to various stressors, including contacts with emotionally demanding patients [13], increased workload and time costs, constantly evolving technologies and institutional and professional ethical challenges [14]. In addition, nurses often face insufficient resources to cope effectively with large labor needs [14]. As a consequence, healthcare workers often experience chronic psychological stress, which may affect up to 71% of nurses [15], leading to mental outcomes, namely, depressive symptoms and somatization [16,17,18,19] and a chronic state of physical and emotional depletion followed by burnout syndrome [20]. Burnout may not only reduce satisfaction with the work carried out and the effectiveness of the work itself [21,22] but also, as a consequence, lower patients’ satisfaction with the quality of medical assistance [23,24]. Also, the relevance of the current problem has been underlined globally after recognizing ‘job burnout’ as a sensitive occupational health problem due to the long-term effects of work-related psychological stress with multiple symptoms [25]. Emotional exhaustion, depersonalization and poor personal accomplishment are three core features accountable for the development of burnout [26]. What is more, the World Health Organization (WHO) has recently identified burnout as ‘a syndrome caused by a long-term perceived psychological stress at the workplace that has not been effectively managed’ [27].

In terms of the relationship between sleep quality and burnout, research has shown that chronic psychological stress may affect sleep quality, leading to insomnia and reduced sleep time (less than 6 h) reported by participants with intense symptoms of burnout syndrome [28]. Therefore, under these circumstances, insomnia [8,29] can serve as a negative potential predictor for quality of life or may even induce the development of burnout [30,31]. However, on the contrary, other studies have identified that burnout does not act on sleep quality in individuals [32]. On the other hand, sleep disorders may depend on long shifts or rotation patterns of shifts [33,34] affecting the expression of burnout itself [35] as a pathological result of a stress-related process distinguished by depersonalization and emotional exhaustion and a paucity of personal accomplishment [26,36]. Finally, evidence shows that sleep quality as a mediator plays a significant role in forecasting the potential impingement of perceived psychological stress upon burnout, both directly and indirectly [37].

Whilst there is public recognition of the need for efforts to reduce burnout among nurses in the clinical environment through interventions to overcome perceived psychological stress and promote healthy sleep hygiene, however, to date, there has been no extensive research on the confirmatory relationship between sleep quality and psychological distress-induced burnout syndrome from the perspective of Lithuanian clinical nurses. Although both sleep disorders and burnout as single variables have been thoroughly researched in recent years, little is known about the association between them. This study not only fills the scientific gap but also brings benefits to provide a theoretical basis for nursing management, improve the quality of sleep and, at the same time, ensure patient safety. This study aimed to explore any possible association between sleep disorders and burnout in a sample of Lithuanian clinical nurses. The following research hypotheses (alternative hypothesis (H_a_) and null hypothesis (H_0_)) were constructed:

**H_a_.**  *Sleep quality risk scores (both the global score and component scores) have an association with burnout symptoms experienced by Lithuanian clinical nurses.*

**H_0_.**  *Sleep quality risk scores (both the global score and component scores) have no relationship with burnout symptoms experienced by Lithuanian clinical nurses.*

## 2. Materials and Methods

### 2.1. Study Design, Population and Data Collection

During a six-week period in October–November 2024, a web-based observational cross-sectional study in design was undertaken in the principal cities of Lithuania, including Panevėžys, Kaunas and Vilnius.

In 2024, the number of practicing and working nurses, including nurse midwives, amounted to 23,160 employees in Lithuanian healthcare institutions [38]. A priori representative sample size (n = 150–378) with a confidence level of 95% and a marginal error of 5–8% was calculated from the target cohort of professional nurses when applying OpenEpi version 3.01 [39]. In terms of the selection procedure, taking into account the geography of the Republic of Lithuania, a partially probabilistic cluster sampling technique was used in the study. According to the main strata in major cities, namely, Vilnius (capital), Kaunas and Panevėžys, the study was designed to pool 6299 and 3012 nurses from 3 urban public healthcare institutions in advance [40,41,42]. However, the automatic involvement of study participants in the study was executed using the websites of nine official social media groups designed and administered for professional nurses in Lithuania. The survey respondents were nurses who navigated to the target online platforms and accessed the target sites through a link from the community manager. Furthermore, according to the design of a cross-sectional study, a total of 18,752 nurses were invited to participate in this observational study. After the nurses had given informed consent and were involved in the study, they were asked to navigate to the internet site and complete a questionnaire. Given that the survey was integrated in an online management system, the web-based E-survey Research Application version 204 (Apklausa, Vilnius, Lithuania) was used to collect the data from nurses [43].

The study participant inclusion criteria were set as follows: (a) qualified and licensed nurses; (b) certified clinical nurse specialists; (c) certified nurse midwives; (d) nurses employed in hospitals of Lithuanian largest cities. Over the course of the study, out of the eligible population of 18,752 clinical nurses, 18,843 study participants were excluded from the study based on the deficiency of inclusion criteria or declining to provide feedback. In this context, the exclusion criteria were constructed as follows: (a) nurses who declined to participate in the study; (b) male nurses (due to the relatively low number of male nurses employed in Lithuanian hospitals); (c) nurses with outdated nursing practices; (d) non-licensed nursing students; (e) nurses hospitalized over one year due to mental disorders.

**Figure 1:**
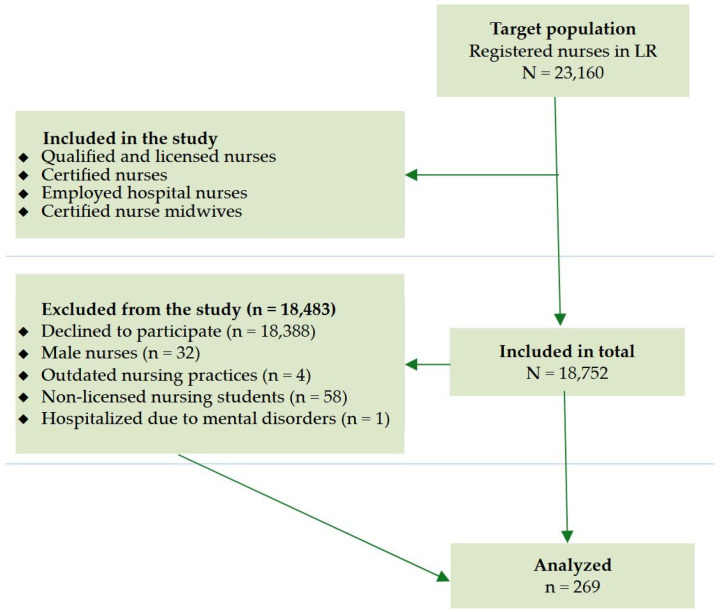
A flowchart of exclusion criteria. LR—Republic of Lithuania.

It should be highlighted that the final response rates of nurses employed in Vilnius, Kaunas and Panevėžys were 2.9% (n = 182) and 2.9% (n = 87), respectively. As a result, the study included and analyzed the data of female nurses (n = 269) aged 22 to 67 within October–November 2024. A more in-depth evaluation of the study enrollment and the selection process is depicted in Figure 1.

### 2.2. Measures

An anonymous, online, self-reported survey for nurses was composed of three subsections. In the first and second sections of the questionnaire, the Pittsburgh Sleep Quality Index (PSQI) tool [44] and the Maslach Burnout Inventory (MBI) [45] were applied to assess the level of subjective sleep quality over the last month and the self-perceived occupational burnout experienced by clinical nurses, respectively.

The PSQI was developed in 1989 by Buysse et al. [44]. The overall internal reliability and validity of the Lithuanian PSQI version was confirmed in Lithuanian populations [46,47], with a Cronbach’s alpha value of 0.7. It should be highlighted that the PSQI was used to measure the subjective sleep quality over the last month. It consisted of 19 items for the study participant. The PSQI items were divided into seven subdimensions reflecting the sleep quality and value as follows: (1) ‘subjective sleep quality’; (2) ‘sleep latency’; (3) ‘sleep duration’; (4) ‘habitual sleep efficacy’; (5) ‘sleep disturbance’; (6) ‘use of sleeping pills’; (7) ‘daytime sleep dysfunction’. Each group was scored from 0 to 3 points (a lower score means a better share of sleep quality). The total score for all seven groups was calculated and the global PSQI score ranged from 0 to 21. In terms of the cut-off point, a score above 5 identified poor sleep quality, while a score equal to or below 5 referred to good sleep quality [44]. Also, sleep quality was further investigated according to the ‘three-factor PSQI model’ suggested by Cole et al. [48]. The sleep quality scale was segmented into three factors depending on the outcomes derived from the confirmatory factor analysis. These factors included the following: (1) Factor 1 (‘sleep efficiency’ related to components of sleep duration and sleep efficiency); (2) Factor 2 (‘sleep quality’ associated with sleep latency, subjective sleep quality and sleep medication use components); (3) Factor 3 (‘daily disturbances’ related to the components of both sleep disorders and daily dysfunction) [49].

The incidence of occupational burnout among nurses was measured by the MBI, developed by Maslach et al. [45], consisting of twenty-two items in three different subdomains, namely, emotional exhaustion (MBI-EE; nine items), depersonalization (MBI-D; five items) and personal accomplishment (MBI-PA; five items). Although emotional exhaustion serves as a precursor to other symptoms of burnout [45], in nursing studies, this subdimension is often applied as a proxy for burnout [50,51,52]. The items for evaluating accomplishment were all positive, in comparison to other subscales. According to the Likert-type scale, all items on the occupational burnout scale were rated from 0 to 6 points where zero points corresponded to ‘never’ and 6 points meant ‘every day’. Overall, high scores obtained from the MBI-EE and MBI-D subdimensions and low (reverse) scores obtained from the MBI-PA subscale indicated a high expression of burnout symptoms. Depending on cut-off points, for the MBI-EE, MBI-D and MBI-PA subscales, a low level range varied from less or equal to 16, 6 and 31 points; a moderate level ranged from 18 to 29, 6 to 11 and 24 to 39 points; and a high level was above 17, 13 and 40 points, respectively. For MBI subscales, the Cronbach alpha values fluctuated from 0.8 to 0.9, indicating good internal consistency and reliability of the instrument used [50].

Given the fact that, in the scientific field, the occurrence of professional burnout among nurses has been associated with sociodemographic factors [19,53,54], the third part of the questionnaire was constructed by the study authors on the basis of the study participants’ sociodemographic and occupational characteristics, namely, sex (male or female), age (in years), number of working hours per week, education levels (with the response alternatives of ‘post-secondary non-tertiary education’, ‘College’ or ‘University’), marital status (with the response choices ‘single and childless’, ‘in a relationship or married’ or ‘divorced’), net average monthly salary (in EUR) (with the response options ‘≤1000 EUR’, ‘1001–2000 EUR’, or ‘≥2001 EUR’), workplace (with response alternatives of ‘an internal medicine unit’, ‘an emergency profile unit’, ‘an intensive care unit’, ‘a surgical profile unit’, ‘a rehabilitation unit’, ‘a neurology unit’ or ‘a cardiac care unit’), seniority status (with the response alternatives of ‘1–5 years’, ‘6–20 years’ or ‘≥20 years’), nursing shifts (with the response choices ‘non-shift worker’ or ‘shift worker’) and employee status (with the response options ‘part-time worker’, ‘full-time worker’ or ‘overtime worker’).

### 2.3. Statistical Data Analysis

The statistical data analysis examined the normality of numerical data using the Shapiro–Wilk test. All categorical data were represented via the contingency tables. The differences in categorical variables (age groups, education and income levels, marital and seniority status, workplace, nursing shifts and the employee profile) between subgroups of the nurses with different levels of the PSQIs were assessed using the chi-square test (*χ*^2^) coupled with odds ratios (ORs).

The measures of central tendency (mean (M) (standard deviation (SD)) were applied to reveal the gross scores of the data under analysis. Before performing the regression analysis, in order to find a possible correlation between confounders and dependent/independent variables, both the two-sample *t*-test coupled with Cohen’s D estimates, which served as the effect sizes (*d*), and the analysis of variance (ANOVA) along with the standardized effect sizes (*η*^2^*_p_*) were used to assess the differences between the mean scores of the MBI-EE, MBI-D and MBI-PA subscales and the significant sociodemographic variables of the study participants. In line with Cohen [55], the exact coefficients were interpreted as follows: ‘a small effect size’ (0.2 ≤ *d* < 0.5), ‘a moderate effect size’ (0.5 ≤ *d* < 0.8) and ‘a large effect size’ (*d* ≥ 0.8). The cut-offs for the qualitative values of *η*^2^*_p_* were interpreted as follows: ‘a small effect size’ (0.01 ≤ *η*^2^*_p_* < 0.06), ‘a medium effect size’ (0.06 ≤ *η*^2^*_p_* < 0.14) and ‘a large effect size’ (*η*^2^*_p_* ≥ 0.14).

The multiple linear regression models were constructed to evaluate the association between burnout symptoms experienced by Lithuanian clinical nurses as a dependent variable and independent variables, namely, the sleep quality risk scores (both the global PSQI score and component scores). For all linear regression models, the confounding variables were set as follows: age, marital status and the duration of nursing experience (in years). In addition, the coefficient of determination (*R*-Squared (*R*^2^)) and F-statistic were calculated to check the goodness of fit of each linear regression model. The critical value of the significance level was fixed as alpha (α) = 0.05 in all statistical tests performed.

The statistical data analysis was carried out using the Statistical Package for the Social Sciences (IBM SPSS Statistics) version 25.0 for Windows (IBM Corp, Armonk, NY, USA). The free and open-source software LibreOffice version 7.6.4 along with SPSS software were adjusted to visualize the statistical data. The cross-sectional study was performed commensurate with the STROBE (strengthening the reporting of observational studies in epidemiology) checklist [56].

## 3. Results

### 3.1. Descriptive and Bivariate Analyses

Two hundred sixty-nine nurses (n = 269) with a mean age of 39.4 ± 1.7 years who reported nursing experience between 1 and 39 years participated in a single cross-sectional study. As indicated in Table 1, the vast majority of study participants were in a relationship or married (74%) and took the level of college (49.1%) or university (34.9%) degrees. All the nurses employed in public healthcare institutions were registered and co-operated with healthcare professionals in the major cities of Lithuania, namely, Vilnius (67.7%), Kaunas and Panevėžys (32.3%). Stemming from the collected data, it was found that the nurses worked at seven hospital departments: an internal medicine unit (30.1%), an emergency profile unit (12.6%), an intensive care unit (17.5%), a surgical profile unit (14.5%), a rehabilitation unit (14.5%), a neurology unit (7.4%) and a cardiac care unit (3.3%). Considering the nature of the nursing shifts, 45% and 55% of the nurses were committed as non-shift workers and shift employees, respectively. More demanding sociodemographic and occupational characteristics of study participants are displayed in Table 1.

**Figure 2:**
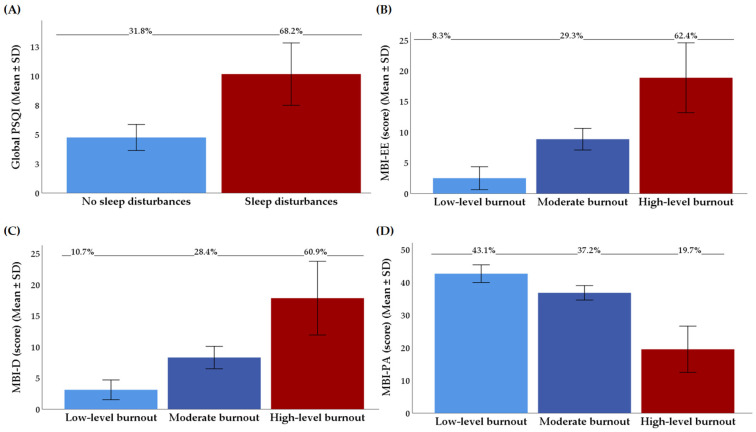
(**A**) Distribution of the results from the Pittsburgh Sleep Quality Index (PSQI); (**B**) distribution of the results from the MBI-E subscale of the MBI; (**C**) distribution of the results from the MBI-D subscale of the MBI; (**D**) distribution of the results from the MBI-PA subscale of the MBI; all data are presented as means ± SD values and percentages (%). The PSQI—the Pittsburgh Sleep Quality Index; MBI-D—depersonalization subscale of the MBI; MBI-EE—emotional exhaustion subscale of the MBI; MBI-PA—personal accomplishment subscale of the MBI; MBI—the Maslach Burnout Inventory; SD—standard deviation.

The total mean of all the PSQI factors was estimated to be 7.2 with a standard deviation of 3.3. Depending on the structure of the three-factor PSQI model, the three-dimensional estimates for Factor 1 (‘sleep efficiency’), Factor 2 (‘sleep quality’) and Factor 3 (‘daily disturbances’) corresponded to 1.5 ± 0.1, 3.3 ± 0.1, and 2.4 ± 0.1 scores, respectively. In agreement with the cut-off of 5 on the PSQI, more than 60% of the nurses were identified as having sleep disturbances (Figure 2A).

Table 1 shows the distribution of nurses (in percentage) by sleep quality depending on the sociodemographic and occupational characteristics. No statistically significant differences were observed between sleep quality and educational and income levels, marital and employee status, workplace, duration of nursing experience or nursing shifts (*p* > 0.05). Alternatively, in agreement with the cut-off of 5 on the PSQI, in early adulthood, 84.3% of the nurses were recognized as having relevant sleep disturbances more frequently compared to the proportion (72.9%) of nurses representing a 40–67-year-old cohort (OR 2.0, 95% CI: 1.3; 3.6, *p* = 0.022).

Figure 2B–D represent the results (means ± SD values and percentages (%)) from the emotional exhaustion, depersonalization and personal accomplishment subscales according to the levels of burnout expression (in terms of ‘low-level burnout’, ‘moderate burnout’ and ‘high-level burnout’). Depending on each burnout domain, 62.4% of nurses had a high score for emotional exhaustion (M _MBI-EE_ score in category: 18.9 ± 5.7), 60.9% had a high level for depersonalization (M _MBI-D_ score in category: 17.8 ± 5.9) and 19.7% had a low level for professional accomplishment (M _MBI-PA_ score in category: 19.5 ± 7.1).

As shown in Table 2, the bivariate analyses disclosed a significant difference between the score of the MBI depersonalization subscale and sociodemographic characteristics, namely, age, marital status and duration of nursing experience in a sample of nurses. More specifically, professional burnout symptoms related to depersonalization were more common in single nurses (*η*^2^*_p_* 0.11, *p* < 0.001) aged between 22 and 39 years (*d* 0.4, *p* = 0.011) with a shorter nursing experience (*η*^2^*_p_* 0.04, *p* = 0.049), i.e., 1–5 years compared to married or divorced study participants aged between 40 and 67 years who had worked in the healthcare system for more than twenty years.

### 3.2. Sleep Quality in Association with Professional Burnout

**Figure 3:**
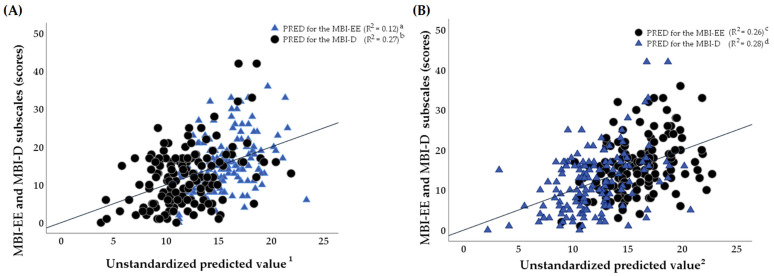
(**A**,**B**) A graphic representation of multiple linear regression models (independent variables: ^1^—Pittsburgh Sleep Quality Index (PSQI), ^2^—Factor 3: daily disturbances (score) (PREDs); dependent variables are the emotion exhaustion (EE) subscale and the depersonalization (D) subscale of the Maslach Burnout Inventory (MBI)). The multiple regression models were adjusted for age, marital status and the duration of nursing experience (in years). (**A**): ^a^—Model (a): F_4.265_ = 4.8, *p* < 0.001, *R*^2^ = 0.12; ^b^—Model (b): F_4.265_ = 8.5, *p* < 0.001, *R*^2^ = 0.27; (**B**): ^c^—Model (c): F_4.265_ = 8.7, *p* < 0.001, *R*^2^ = 0.26; ^d^—Model (d): F_4.265_ = 9.2, *p* < 0.001, *R*^2^ = 0.28. See Table 3 for further details. F—the F-statistic, PRED—unstandardized predicted value, *R*^2^—*R*-Squared.

Figure 3A,B display multiple linear regression analyses that were represented based on the predicted values (PREDs) for the intensity of perceived professional burnout emanating from a linear combination of the potential predictors, namely, different domains of the three-factor PSQI, as follows: Factor 1 (‘sleep efficiency’), Factor 2 (‘sleep quality’) and Factor 3 (‘daily disturbances’). In the multiple linear regression analysis, the confounders as covariates were set as follows: age, marital status and the duration of nursing experience (in years). However, the covariates that were not recruited as potential confounders were those such as nursing shifts, workload, education levels, average monthly net salary, workplace and employee status, which, during the bivariate analysis process, were not correlated with burnout symptomatology or sleep quality in a sample of clinical nurses.

According to Table 3, although the global PSQI score was related to the estimates of both the emotion exhaustion (β 0.6, 95% CI 0.3; 0.9, *p* < 0.001, F_4.265_ = 6.1, *R*^2^ = 0.12) and depersonalization (β 0.5, 95% CI 0.2; 0.9, *p* = 0.002, F_4.265_ = 8.1, *R*^2^ = 0.27) MBI subscales, the *R*-Squared values identified that the study findings did not fit the linear regression model (in terms of *R*^2^ < 25%) as well as not being suitable for explaining the PSQI values and the global PSQI’s predictive role for the potential development of emotional exhaustion in clinical nurses.

Additionally, the only Factor 3 referring to daily disturbances as one of the PSQI sub-dimensions was associated with MBI subscales in a double mode in a sample of clinical nurses. More specifically, the higher levels of both emotion exhaustion (β 2.5, 95% CI 1.5; 3.5, *p* < 0.001, F_4.265_ = 8.7, *R*^2^ = 0.26) and depersonalization (β 1.9, 95% CI 0.8; 3.0, *p* = 0.001, F_4.265_ = 8.2, *R*^2^ = 0.28) were predicted by perceived daily disturbances (in terms of sleep disturbances and daytime dysfunction) in nurses.

## 4. Discussion

### 4.1. Sleep Quality and Burnout Symptomatology Proportions

According to the study, 68.2% of Lithuanian nurses experienced poor sleep quality. The data referring to sleep disorders were confirmed by the global PSQI, which equaled a score of 7.2 ± 3.3. The empirical data of our study were consistent with the meta-analysis of the fifty-three observational studies reviewed by Zeng et al. [57] that revealed an elevated risk for the development of sleep disorders in 61% of nurses (PSQI 7.1 ± 0.18 score). However, in Lithuanian nurses, the negative trend was observed when the global PSQI values ranging from 4.9 to 6.4 were compared to the PSQIs of Italian [21], Spanish [58], Japanese [59], Polish [60] and Latvian [61] nurses. In contrast, the significantly higher proportion of nurses dealing with abnormal sleep behaviors was reported in Turkey [62] and South Korea [16] (7.3 < PSQI < 9.7).

This cross-sectional study revealed the expression of occupational burnout in a cohort of Lithuanian female nurses. The high levels of emotional exhaustion, depersonalization and lack of personal accomplishment were experienced by 62.4% (M _MBI-EE_ score: 15.7), 60.9% (M _MBI-D_ score: 11.8) and 19.7% (M _MBI-PA_ score: 23.2) of nurses we studied, respectively. It should be noted that higher rates of emotional exhaustion were established among nurses from other European Union countries such as Greece [63,64], Poland, Germany, Italy and Hungary [65]. Meanwhile, a similar level of emotional exhaustion (MMBI-EE score fluctuated from 12.9 to 19.0) was observed in nurses working in Spain [66], France [67], the UK [68], the Czech Republic and Slovakia [65].

It should be highlighted that, in the Lithuanian nurses we researched, the level of depersonalization component development was relatively high in comparison with the depersonalization estimates observed in nurses from Spain [66], Greece [63,64], France [67], the UK [68], Italy, Hungary and the Czech Republic [65] (M _MBI-D_ score: 12 vs. 8). On the other hand, the expression of depersonalization among Lithuanian nurses was consistent with the similar values detected among nurses working in hospitals in geographically closer neighboring countries, namely, Poland and Germany [65].

Although this cross-sectional study revealed that sleep quality was a potential factor for the development of occupational burnout in Lithuanian nurses, in the regression analysis, the controlled confounders, namely, age, marital status and working experience in hospital-based care, were significant, too. In the early stages of this study, we expected that age would be negatively associated with the subscales of both emotional exhaustion and depersonalization as well as having a positive relation to the personal accomplishment domain of burnout in a sample of clinical nurses. Based on our study results, only a one-way relationship was identified between nurses’ younger age (i.e., younger nurses with 1–5 years’ seniority) and high-level depersonalization. However, age was not related to emotional exhaustion. In this context, given that age may affect occupational burnout and depersonalization among nurses, the association is complex and sometimes conflicting, with some research finding that younger nurses were at an increased risk of emotional exhaustion and depersonalization, while other studies claimed that older nurses with more working experience were more exposed to depersonalization [53,69]. Also, we have hypothesized that the age-related facet of depersonalization can be explained by the fact that findings derived from previous research have confirmed that the age of nurses was negatively correlated with both the maladaptive coping style [70] and depersonalization [69]. Additionally, our study has shown that being single and non-parental is related to the higher levels of the depersonalization score. These data may be explained by the fact that the family environment in which the couple lives serves as a vector that ensures security and social support, and which prevents the individual from developing cynical, detached and negative attitudes towards fellow workers. These findings correspond with those reported by other researchers [54,71,72]. Moreover, the obligation to raise children does not appear to exacerbate but rather reduces the emotional congestion and feeling of overworking that nurses often undergo [53].

### 4.2. Association Between Sleep Quality and Burnout

**Figure 4:**
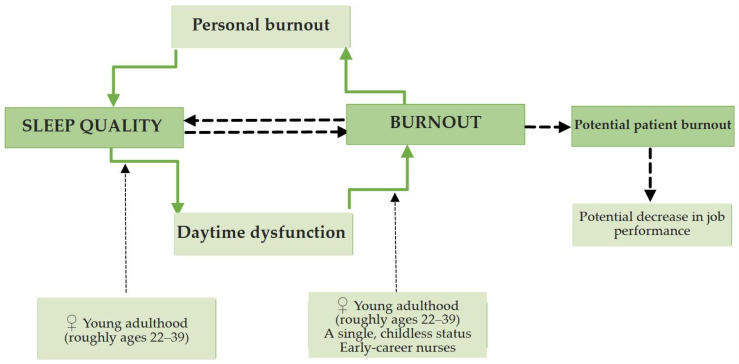
A graphic representation of the conceptual framework derived from the study data and considering the bidirectional relationship between sleep quality and burnout symptomatology in clinical nurses. ♀—females.

In terms of the three-factor PSQI scoring model, the sample of Lithuanian nurses has demonstrated the association of sleep quality with the symptomatology of burnout syndrome, however, in a specific manner. More specifically, the global PSQI estimate has related only to higher levels of depersonalization experienced by nurses. In the scientific literature, we have found analogous results that can explain the principle of this relationship. Although the relationship between sleep disorders and burnout symptomatology could be interpreted in a bidirectional way (Figure 4), the findings obtained from this study were consistent with the research conducted into medical personnel [21,73,74] confirming that poor sleep quality as a potential risk factor was positively associated with burnout.

This might be due to the fact that burnout, established on Conservation of Resources Theory [75], arises from a permanent loss of resources, which people do not have the ability to replenish, while sleep alone can help to halt the spiral of loss of these resources and contribute to the retention of other resources (e.g., good job performance) [74]. The disturbance of the hypothalamic–pituitary–adrenal (HPA) axis, which is generally adhered to both humans reporting burnout and those with sleep disorders, could partially shed light on the association persisting between sleep quality and burnout [74,76]. It has been implied that poor sleep quality is related to a hyperactive condition that is also an integral component of burnout and may lead to an intensified activation of the HPA axis, generating a rise in an escalating trend in allostatic load. Thus, the intensified activation of the HPA axis, which is the central strain feedback system and accountable for the building of resilience (in terms of long-term stress adaptation), might contribute to an intermediary role in the association between sleep disturbance and burnout [77]. Another clarification could be that sleep disturbances are closely linked to long-term cognitive hyperstimulation, or the incapability to destress or disengage from thoughts related to work during spare time, which has also been found to result in burnout expression [78]. Therefore, an insufficient recovery pathway was shown to fluctuate from labor demands to sleep disturbances and, in the long term, to the development of burnout syndrome [78].

Furthermore, the sample of nurses under analysis has shown a close interlink between all three core components of occupational burnout although the global PSQI score did not significantly predict the expression of emotional exhaustion. In particular, emotional exhaustion is not only the leading cause of burnout syndrome but also results in depersonalization, leading to a restriction in personal accomplishment [19,50,79,80]. Therefore, it can be assumed that sleep disorders may act as a trigger for a full-blown depersonalization in nurses and, at later stages, result in job dissatisfaction and poor quality of nursing care. Furthermore, the study has identified a significant association between ‘daily disturbances’ (in terms of sleep disturbances and daytime dysfunction components) and more exaggerated both emotional exhaustion and depersonalization in a cohort of clinical nurses. Concurrently, the PSQI components, namely, ‘sleep efficiency’ or ‘sleep quality,’ have not revealed a significant relationship with full-blown burnout syndrome among Lithuanian nurses. The present findings indicate the potential benefits of a three-dimensional evaluation of sleep quality in scoring the three factors’ availability because more accurate information on the type and the nature of sleep disorders will facilitate healthcare providers to focus on the selection of treatment [81]. Nevertheless, preventive interventions should serve as the primary choice strategies. It is well documented that cultural, organizational and systemic factors can shape burnout through high workloads, a lack of control and support, poor leadership and misaligned values, which can all lead to employee stress, exhaustion and disengagement. On the contrary, positive cultures with clear communication, sufficient resources and opportunities for professional development can prevent emotional exhaustion and burnout. In this regard, when the scientific findings indicate a number of possible objectives for the intervention at different organizational levels that could help to find more effective ways of reducing burnout symptomatology and improving work engagement [82], clinical nurses, as vital healthcare professionals, can derive benefit not only from interventions at the individual level but also at the organizational level.

### 4.3. Strengths, Limitations and Further Directions

It should be highlighted that this study was focused on a highly relevant population (in terms of clinical nurses), a group exposed to high occupational demands and working shifts. To our knowledge, this is the first study capable of relating poor sleep hygiene skills with the expression of burnout symptomatology in a population of nurses working in the Baltic States. Secondly, the results of this study can contribute to more effective management of risks affecting the mental health of nurses in the healthcare sector. For example, poor sleep quality remains a vital health issue for hospital staff nurses, and in-service education on sleep hygiene knowledge and skills to implement healthy sleep habits may assist in reducing both sleep disturbances and daytime dysfunction. Moreover, it serves as a moderator for reducing perceived chronic psychological stress coupled with occupational burnout.

Several limitations of this study should be taken into account when summarizing the conclusions. First of all, given that the cross-sectional design of the study allowed for the identification of associations and at the same time restricted the opportunity to establish causation between independent and dependent variables, this observational study was related to the fact that the causal relationship between sleep quality and professional burnout should be assessed with caution. The next limitation was based on a relatively small, yet representative sample size studied and an exclusive reliance on study participants’ self-report measures, which could introduce possible biases (e.g., recall or social desirability), even if the subjects’ outcomes were anonymous. Also, the studied cohort was derived from three favored large cities in Lithuania, which makes it impossible to generalize the study results for some samples of Lithuanian nurses (e.g., nurses working in cultural regions of Lithuania, namely, Samogitia). Thirdly, given males take up the nursing profession especially rarely in Lithuania, including that male nurses may present different sleep quality or sleep–burnout dynamics, our study was restricted in terms of the assessment of the distribution of analytical variables depending on sex. A fortiori, research results published by other authors have revealed a significant correlation between sex and depersonalization [19,83]. Fourthly, according to these limitations, the results of this study can be considered as a starting point for further observational studies (case–control, longitudinal or cohort studies) on the efficiency of possible social support interventions in managing both sleep quality and burnout symptoms perceived by nurses. Also, further additional qualitative research could be beneficial in order to highlight similarities or divergences, and consider how cultural, organizational or systemic factors may shape sleep quality and burnout among the populations of clinical nurses.

In addition, in the absence of consensus on the cut-offs for PSQI scores defining the threshold for poor sleep quality, the threshold for PSQI scores (≥5 points) established in clinical practice and associated with poorer sleep quality as well as an increased likelihood of the development of sleep disorders was used in this study [44,84]. However, due to occupational characteristics such as rotating shifts, irregular sleep–wake schedules and high job-related stress, clinical nurses tend to show relatively higher PSQI scores even among otherwise healthy individuals. Recent studies have reported that applying a higher threshold, such as a cut-off of 6 or 7, may more accurately distinguish poor sleep quality among healthcare workers and shift-working populations. Therefore, it should be noted that further research on sleep quality should verify the PSQI cut-off value when conducting cross-sectional studies involving clinical nurses.

Finally, according to some research [85,86], socio-economic difficulties related to workload and psychological stress exposure can independently contribute to poor sleep quality; therefore, it would be useful to conduct cross-sectional studies in nurses’ populations in order to confirm or decline this induction hypothesis.

## 5. Conclusions

This study has highlighted the worrying proportion of nurses at an increased risk of occupational burnout syndrome after more than three-fifths of nurses experienced the symptoms of emotional exhaustion and depersonalization. A similar proportion of nurses was exposed to the risk of sleep disorders, which, as a potential trigger, played an important role in maintaining burnout syndrome. Thus, further attention of healthcare professionals should be focused on reducing the high-level depersonalization expression and the potential risk factors, namely sleep disturbances and daytime dysfunction, associated with this burnout symptom in a population of clinical nurses. Therefore, by improved and targeted integration of efficient sleep interventions [87], healthcare institutions could promote an employee-friendly workplace, and, eventually, improve not only indicators of burnout syndrome but also the performance of nurses and the safety of patients as well as mutual satisfaction with perceived nursing care.

In addition, the sociodemographic characteristics were related to the burnout syndrome experienced by clinical nurses. More particularly, depersonalization as a facet of burnout was identified as having a higher rank in single, childless nurses 22–39 years of age, who had less than 5 years of occupational experience. These variables ought to be considered when designing risk profiles for nurses. This would contribute to the implementation of health promotion programs or mindfulness therapies for the groups of nurses who are prone to full-blown burnout, and in this fashion, some of its more severe mental outcomes could be avoided.

## Figures and Tables

**Table 1:** The categorization of nurses with different levels of sleep quality depending on the sociodemographic and occupational traits (n = 269).

Variables	Total		PSQI				*χ*^2^ (*p*)	OR [95% CI: LB; UB]
			Good Sleeper (Score: 0–5)		Poor Sleeper (Score: ≥5)			
	N	%	n	%	n	%		
Age groups								
22- to 39-year-old	140	52.0	22	15.7	118	84.3	5.2 (0.022)	2.0 [1.3; 3.6]
40- to 67-year-old	129	48.0	35	27.1	94	72.9		1
Education levels								
Post-secondary non-tertiary	43	16.0	11	25.6	32	74.4	1.7 (0.436)	0.6 [0.3; 1.4]
College	132	49.1	30	22.7	102	77.3		0.7 [0.4; 1.4]
University	94	34.9	16	17.0	78	83.0		1
Marital status								
Single and childless	42	15.6	7	16.7	35	83.3	0.8 (0.677)	1.7 [0.5; 5.4]
In a relationship or married	199	74.0	43	21.6	156	78.4		1.2 [0.5; 3.0]
Divorced	28	10.4	7	25.0	21	75.0		1
Average monthly net salary (in Euro (EUR))								
≤1000 EUR	57	21.2	12	21.1	45	78.9	0.9 (0.649)	0.7 [0.2; 2.1]
1001–2000 EUR	179	66.5	40	22.3	139	77.7		0.6 [0.2; 1.7]
≥2001 EUR	33	12.3	5	15.2	28	84.8		1
Workplace								
Internal medicine unit	81	30.1	20	24.7	61	75.3	7.5 (0.273)	0.4 [0.1; 3.2]
Emergency profile unit	34	12.6	5	14.7	29	85.3		0.7 [0.1; 7.1]
Intensive care unit	47	17.5	14	29.8	33	70.2		0.3 [0.1; 2.6]
Surgical profile unit	39	14.5	7	17.9	32	82.1		0.6 [0.1; 5.3]
Rehabilitation unit	39	14.5	9	23.1	30	76.9		0.4 [0.1; 3.8]
Neurology unit	20	7.4	1	5.0	19	95.0		2.4 [0.1; 42.8]
Cardiac care unit	9	3.3	1	11.1	8	88.9		1
Duration of nursing experience								
1–5 years	104	38.7	20	19.2	84	80.8	1.9 (0.392)	1.5 [0.8; 3.1]
6–20 years	90	33.5	17	18.9	73	81.1		1.6 [0.8; 3.3]
≥20 years	75	27.9	20	26.7	55	73.3		1
Nursing shifts								
Non-shift work	121	45.0	24	19.8	97	80.2	0.2 (0.623)	1.2 [0.6; 3.1]
Shift work	148	55.0	33	22.3	115	77.7		1
Number of working hours per week								
36–40 h	92	34.2	20	21.7	72	78.3	1.4 (0.487)	1.2 [0.6; 2.4]
41–50 h	92	34.2	16	17.4	76	82.6		1.6 [0.8; 3.2]
51–60 h	85	31.6	21	24.7	64	75.3		1
Employee status								
Part-time work	14	5.2	3	21.4	11	78.6	0.1 (0.970)	1.1 [0.2; 3.9]
Full-time work	149	55.4	31	20.8	118	79.2		1.1 [0.5; 1.9]
Overtime work	106	39.4	23	21.7	83	78.3		1

The PSQI—the Pittsburgh Sleep Quality Index; *χ*^2^—the chi-squared test; OR—odds ratio; 95% CI—95% confidence interval; LB—lower bound; UB—upper bound; *p*—*p*-value.

**Table 2:** Distribution of Maslach Burnout Inventory scores depending on various descriptive characteristics of nurses.

Variables	Maslach Burnout Inventory		
	Emotional Exhaustion	Depersonalization	Personal Accomplishment
Age groups			
22- to 39-year-old	16.3 ± 7.3	13.1 ± 8.1	22.6 ± 9.1
40- to 67-year-old	15.2 ± 6.5	10.2 ± 6.7	24.2 ± 11.2
*d* ^1^	0.2	0.4	−0.1
*p*	0.225	0.011	*p* = 0.456
Marital status			
Single and childless	17.6 ± 7.9	17.6 ± 10.6	21.9 ± 11.5
In a relationship or married	15.6 ± 6.8	11.1 ± 6.6	23.1 ± 9.7
Divorced	14.8 ± 7.1	8.8 ± 6.5	28.6 ± 10.4
*η*^2^*_p_* ^2^	0.02	0.11	0.01
*p*	0.267	<0.001	0.562
Duration of nursing experience			
1–5 years	16.4 ± 7.1	13.1 ± 8.7	23.1 ± 9.8
6–20 years	16.4 ± 7.2	11.9 ± 6.1	23.2 ± 9.8
≥20 years	14.3 ± 6.6	9.7 ± 6.5	23.6 ± 10.9
*η*^2^*_p_* ^2^	0.03	0.04	<0.01
*p*	0.163	0.049	0.995
Total score	15.7 ± 7.1	11.8 ± 7.7	23.2 ± 10.1

All data are presented as means ± SD values. ^1^—the *t*-test coupled with Cohen’s D (*d*) effect sizes; ^2^—the analysis of variance (ANOVA) expressed with the standardized effect size (*η*^2^*_p_*); *p*—*p*-value.

**Table 3:** The association between the individual factors of the Pittsburgh Sleep Quality Index (three-factor model) and professional burnout as a dependent variable in a sample of nurses (multiple regression analyses).

Model	Independent Variable	β	95% CI [LB; UB]	*p*	F_4.265_	*R* ^2^
1.1. MBI-EE (score) ^a × 1^	PSQI (score) ^1^	0.6	[0.3; 0.9]	<0.001	6.1	0.12
1.2. MBI-EE (score) ^a × 2^	Factor 1: SE (score) ^2^	0.1	[–0.7; 0.8]	0.924	1.7	0.18
1.3. MBI-EE (score) ^a × 3^	Factor 2: SQ (score) ^3^	0.2	[–0.5; 0.9]	0.611	3.4	0.18
1.4. MBI-EE (score) ^a × 4^	Factor 3: DD (score) ^4^	2.5	[1.5; 3.5]	<0.001	8.7	0.26
2.1. MBI-D (score) ^b × 1^	PSQI (score) ^1^	0.5	[0.2; 0.9]	0.002	8.1	0.27
2.2. MBI-D (score) ^b × 2^	Factor 1: SE (score) ^2^	0.6	[–0.2; 1.3]	0.151	6.1	0.19
2.3. MBI-D (score) ^b × 3^	Factor 2: SQ (score) ^3^	0.1	[–0.7; 0.8]	0.939	5.6	0.19
2.4. MBI-D (score) ^b × 4^	Factor 3: DD (score) ^4^	1.9	[0.8; 3.0]	0.001	8.2	0.28
3.1. MBI-PA (score) ^c × 1^	PSQI (score) ^1^	–0.1	[–0.5; 0.5]	0.861	0.3	0.01
3.2. MBI-PA (score) ^c × 2^	Factor 1: SE (score) ^2^	0.4	[–0.7; 1.5]	0.430	0.4	0.01
3.3. MBI-PA (score) ^c × 3^	Factor 2: SQ (score) ^3^	–0.6	[–1.5; 0.3]	0.221	0.7	0.02
3.4. MBI-PA (score) ^c × 4^	Factor 3: DD (score) ^4^	0.3	[–1.2; 1.9]	0.684	0.4	0.01

^a–c^—dependent variables for multiple linear regression models. ^1–4^—independent variables (in terms of three-factor Pittsburgh Sleep Quality Index (PSQI)) for multiple linear regression models. All regression models were adjusted for age, marital status and the duration of nursing experience (in years). MBI-D—depersonalization subscale of the MBI; MBI-EE—emotional exhaustion subscale of the MBI; MBI-PA—personal accomplishment subscale of the MBI; Factor 1: SE—Factor 1: sleep efficiency; Factor 2: SQ—Factor 2: sleep quality; Factor 3: DD—Factor 3: daily disturbances; F—F-statistic; 95% CI—95% confidence interval; LB—lower bound; UB—upper bound; *R*^2^—the *R*-Squared; MBI—the Maslach Burnout Inventory; *p*—*p*-value.

## Data Availability

Raw data obtained from this study are not publicly accessible because sharing them would go against ethical considerations, such as study participants’ privacy and consent; however, researchers can still request access to the research data from the corresponding author, often under specific conditions.

## Abbreviations

ANOVA	Analysis of variance
CI	Confidence interval
D	Depersonalization
EE	Emotional exhaustion
EUR	Euro
LR	Republic of Lithuania
M	Mean
MBI	Maslach Burnout Inventory
PA	Personal accomplishment
PSQI	Pittsburgh Sleep Quality Index
SD	Standard deviation
WHO	World Health Organization
